# A way forward for fundamental physics in space

**DOI:** 10.1038/s41526-022-00229-0

**Published:** 2022-11-02

**Authors:** A. Bassi, L. Cacciapuoti, S. Capozziello, S. Dell’Agnello, E. Diamanti, D. Giulini, L. Iess, P. Jetzer, S. K. Joshi, A. Landragin, C. Le Poncin-Lafitte, E. Rasel, A. Roura, C. Salomon, H. Ulbricht

**Affiliations:** 1 Department of Physics, University of Trieste, Strada Costiera 11, 34151 Trieste, Italy; 2 Istituto Nazionale di Fisica Nucleare, Trieste Section, Via Valerio 2, 34127 Trieste, Italy; 3 European Space Agency, Keplerlaan 1 - P.O. Box 299, 2200 AG Noordwijk, ZH The Netherlands; 4 Dipartimento di Fisica ‘E. Pancini’, Università di Napoli ‘Federico II’, INFN, Sezione di Napoli, via Cinthia 9, I-80126 Napoli, Italy; 5 Scuola Superiore Meridionale, Largo S. Marcellino 10, I-80138 Napoli, Italy; 6 Istituto Nazionale di Fisica Nucleare, Laboratori Nazionali di Frascati (INFN-LNF), via E. Fermi 54, 00044 Frascati (Rome), Italy; 7 LIP6, CNRS, Sorbonne Université, Paris, France; 8 Institute for Theoretical Physics, Leibniz University Hannover, Appelstrasse 2, 30167 Hannover, Germany; 9 Sapienza Università di Roma, 00184 Rome, Italy; 10 Department of Physics, University of Zürich, Winterthurerstrasse 190, 8057 Zürich, Switzerland; 11 Quantum Engineering Technology Labs, H. H. Wills Physics Laboratory & Department of Electrical and Electronic Engineering, University of Bristol, Bristol, UK; 12 SYRTE, Observatoire de Paris, Université PSL, CNRS, Sorbonne Université, LNE, Paris, France; 13 Leibniz Universität Hannover, Institut für Quantenoptik, Welfengarten 1, 30167 Hannover, Germany; 14 Institute of Quantum Technologies, German Aerospace Center (DLR), Wilhelm-Runge-Straße 10, 89081 Ulm, Germany; 15 Laboratoire Kastler Brossel, ENS-Université PSL, CNRS, Sorbonne Université, Collège de France, Paris, France; 16 School of Physics and Astronomy, University of Southampton, SO17 1BJ, Southampton, United Kingdom

**Keywords:** Atomic and molecular physics, Condensed-matter physics

## Abstract

Space-based research can provide a major leap forward in the study of key open questions in the fundamental physics domain. They include the validity of Einstein’s Equivalence principle, the origin and the nature of dark matter and dark energy, decoherence and collapse models in quantum mechanics, and the physics of quantum many-body systems. Cold-atom sensors and quantum technologies have drastically changed the approach to precision measurements. Atomic clocks and atom interferometers as well as classical and quantum links can be used to measure tiny variations of the space-time metric, elusive accelerations, and faint forces to test our knowledge of the physical laws ruling the Universe. In space, such instruments can benefit from unique conditions that allow improving both their precision and the signal to be measured. In this paper, we discuss the scientific priorities of a space-based research program in fundamental physics.

## Introduction

The Standard Model (SM) of particle physics and General Relativity (GR) are the two pillars of our current understanding of Nature. Both theories have been probed individually with ever-increasing precision and are consistent with nearly all experimental observations. However, they fail to explain dark matter (DM), dark energy (DE), or the imbalance between matter and anti-matter in the universe. Yet DM and DE represent 95% of the energy content of our universe while known matter (atoms, molecules) amounts to only 5%. Today, DM and DE have an unknown origin and there is a great deal of experimental and theoretical activity to solve this puzzle. In summary, the clustering of large-scale structures and the accelerated behavior of cosmic fluid could be addressed whether finding out new (unknown) forms of matter or assuming that gravity behaves in a different way at infrared scales. Furthermore, the lack of a self-consistent theory of Quantum Gravity prevents the unification of SM and GR at ultraviolet scales.

The open questions in fundamental physics investigated in this paper are:

- Validity of Einstein’s Equivalence Principle;
- Origin and nature of dark matter and dark energy;
- Decoherence and collapse models in quantum mechanics;
- Quantum many-body physics.

They will be addressed from different research corners and with different experimental methods:

- Ultracold atoms;
- High-stability and -accuracy atomic clocks;
- Matter-wave interferometry;
- Classical and quantum links.

In view of these issues, the cosmos is a particularly attractive laboratory as it provides particles (cosmic rays) or objects (black holes, neutron stars) which are not produced in man-made laboratories (Even if very relevant to fundamental physics, gravitational waves are not discussed here as already addressed in a parallel paper on astrophysics research in space.).

## Open problems in fundamental physics

### Einstein’s Equivalence Principle

The Equivalence Principle (EP) is at the foundation of Einstein’s GR. It states the universal coupling of matter to the gravitational field, which in turn implies that the impact of gravity onto matter can be understood in terms of a common geometric structure of space-time. The relevance of EP is twofold: First, it clearly goes beyond GR and will serve as a decisive tool for discriminating competing theories of gravity^1^. Second, understanding its role and impact for couplings to the genuine quantum matter will be a first and decisive step in probing the interface between Quantum Theory and GR in a way guided by experiments, with possible far-reaching implications as regards possible reconciliations of the incompatible foundations of these theories.

The first formulation of EP, also known as the Weak Equivalence Principle (WEP), states the universality of free fall (UFF), which is meant to say that the center-of-mass motion of a sufficiently unstructured test body only depends on the initial conditions and not on the details of body’s further constitution. In a Newtonian setting, this is sometimes stated as the strict equality of body’s inertial mass *m*_*i*_ with its gravitational mass *m*_*g*_, though these two concepts of masses do not easily generalize to other frameworks outside Newtonian physics. The consequences of UFF include the impossibility of locally distinguish effects due to a gravitational field from those arising in a uniformly accelerated reference frame^2^. For strictly uniform gravitational fields this pertains to ordinary Quantum Mechanics (QM)^3^. Generally, this entails that it is always possible to locally describe the first-order neighborhood of any space-time point with the language of Special Relativity. This is a crucial aspect; indeed, in 1920 Einstein himself addressed the EP as *"the happiest thought of my life”*^4^, (p. 265).

Today the general formulation of the EP, known as the *Einstein Equivalence Principle*, comes in three parts, of which the universality of free fall is but one. The complete set of demands comprised in EP read as follows:

- Universality of free fall (WEP) holds;
- The result of any local non-gravitational test does not depend on the velocity of the free-falling experimental apparatus (Local Lorentz Invariance);
- The result of any local non-gravitational test does not depend on where and when in the Universe it was carried out (Local Position Invariance). This last part is also related to the universality of gravitational redshift (UGR) and the universality of clock rates (UCR).

By “local non-gravitational test”, we mean an experiment that takes place in a sufficiently small region of a free-falling laboratory so that tidal effects (i.e., gradients of the gravitational field over distances of the extent of the test body) become negligible. Moreover, this statement of EP assumes that one may neglect gravitational self-interactions of the size of the systems used to probe the external gravitational field. In order to account for modifications or extensions of Einstein gravity, there is the need to introduce an even more general concept, which includes both the previous principles in a suitable limit. Such a requirement results in the *Strong Equivalence Principle* (SEP) which can be formulated as follows:

- WEP is valid for self-gravitating bodies with appreciable fractions of gravitational binding energies contributing to their overall energy, as well as for test bodies;
- The result of any local test neither depends on the velocity of the freely falling experimental apparatus, nor on the position and the time in which it is carried out.

Clearly, the SEP reduces to the Einstein EP in the limit in which the gravitational self-field of the probing body is negligible as regards its strength compared to the external field and also as regards its contribution to the total energy budget of the test body.

These considerations, however, work for WEP, not only in the context of GR, but also for its generalizations and modifications, most importantly, scalar-tensor- and higher-order-theories of gravity. This is true in the metric formulation of several Extended Theories of Gravity, of which GR is a particular case. For a review, we refer to ref. ^5^ and ref. ^6,7^ for applications. As it is well known, violation of EP may arise in scalar-tensor theories. In particular, at a fundamental level temperature may play a crucial role. Indeed, at zero temperature, EP still holds due to the fact that contributions to *m*_*i*_ and *m*_*g*_ that may allow *m*_*g*_/*m*_*i*_ ≠ 1 will vanish as soon as *T* → 0. This result can be shown by employing different approaches, though, in any case, the evaluation of radiative corrections will require techniques from Quantum Field Theory^8^.

### DM and DE

Astrophysical and cosmological observations have established^9^ that DM and DE are the dominant contributions to the average energy density of the universe. However, the precise nature of DM and DE remains elusive despite considerable efforts in observational astrophysics and experimental high-energy physics over several decades. Precision measurements based on atom interferometry and atomic clocks in space can make an important contribution to this quest.

Extensive searches^10^ for massive dark-matter candidates known as WIMPs (weakly interacting massive particles) have come empty-handed, spurring growing experimental interest in exploring a wider range of dark-matter hypotheses. In this respect, the possibility that DM could be attributed to coherent oscillations within subgalactic regions of ultralight scalar (or pseudoscalar) fields has recently been gaining increasing attention^11^. These oscillations can lead to small periodic variations in space and time of the parameters of the Standard Model, which could be detected in highly sensitive gravimetry measurements as a small modulation in the time of the acceleration experienced by freely falling atoms^12^. They can also produce small oscillations of the transition energies between electronic states that could be identified by comparing different atomic clocks at the same location^13^ or pairs of identical atom interferometers separated by long distances but interrogated by common laser beams^14^.

On the other hand, certain classes of dark-energy theories, known as chameleon-field^15^ and symmetron-field^16^ models, involve a light scalar field that can mediate a long-range interaction and give rise to a "fifth force”. However, through its interaction with matter, the chameleon and the symmetron fields acquire a much larger effective mass in any region where the matter density is not too low. This fact leads to a screening of the interaction, which can in this way evade tests of the EP with macroscopic masses. In contrast, microscopic test masses, such as the atoms in a vacuum chamber, are hardly affected by the screening mechanism, as opposed to the source mass.

### Decoherence and collapse models in QM

QM is grounded in the superposition principle, which has been verified in a variety of experiments with microscopic and mesoscopic systems. However, its applicability (therefore, the validity of quantum theory) to macroscopic objects poses a problem, as first exemplified by Schrödinger with the cat paradox^17^: simply, we do not experience quantum superpositions in our daily life, in spite of the fact that - mathematically speaking - they easily carry over from the microscopic world to the macroscopic one.

One way out is to assume that the quantum superposition principle has a limited range of validity: it is not a fundamental principle of nature and progressively breaks down when atoms glue together to form larger and more complex systems^18–23^. Models of spontaneous wavefunction collapse^24,25^ translate this idea into mathematical models: the Schrödinger equation is modified by adding nonlinear and stochastic terms, which induce the collapse of the wavefunction in space. As such, these models predict that systems progressively lose quantum coherence and behave classically; the larger the system, the faster the transition from a quantum to a classical state.

Such a potential loss of coherence can be tested by suitable interferometric techniques. The basic idea is to create a quantum superposition of a system, which is as massive as possible, make it last as long as possible, and then check whether the two branches interfere. If they do, then the quantum theory is right, otherwise, there is a conflict with the theory. The difficulty is to make the superposition, which requires free evolution, last long enough. Here space helps by ensuring much longer free-evolution times than on the ground (see “Matter-wave interferometry tests of GR and QM”).

Recently, alternative and stronger tests have been developed, which are non-interferometric, because they do not require creating a superposition state. They are based on the fact that, according to collapse models, the collapse of the quantum wavefunction is triggered by a noise, which also makes particles diffuse. Then, one can test this diffusion process, which takes place also when systems are not in a spatial superposition^26^. Monitoring the diffusion of the center of mass of a system, or the expansion of a gas of particles, has already placed strong bounds on the collapse parameters^27–31^. Space again helps thanks to the longer free-evolution times (see “Ultracold atom physics in space”).

### Quantum many-body physics

Atoms cooled by laser light followed by evaporative cooling reach ultracold temperatures in the sub-nanokelvin range with an average speed of 10–100 μm/s. In the last two decades, quantum many-body physics with ultracold atoms has experienced spectacular growth with Bose-Einstein condensates (BEC) and superfluid Fermi gases. Many-body physics has entered a new era where quantitative comparisons can be made between theory and experiments. If mean field theories can be successfully used in some weakly interacting systems, the case of strongly correlated bosons or fermions represents today an outstanding challenge. This covers several fields of physics ranging from QCD, condensed matter, astrophysics (neutron stars), nuclear and atomic physics. For ultracold atoms, the Earth’s gravity becomes a major perturbation; free atoms fall! If atoms are confined, compensating gravity imposes limitations on the type of traps that must be used and, as a consequence, on the type of physical phenomena that can be explored. Microgravity platforms offer the appealing possibility to overcome this limitation and access new regimes in ultracold atom many-body physics.

## Need for space

Space provides the ideal conditions for testing fundamental physics. Indeed, a space-based laboratory can ensure long free-fall conditions and long interaction times, important for precision tests where long-duration measurements are needed to average down the noise and characterize the instrument accuracy. Experiments using test masses (macroscopic or atoms) as probes of the space-time metric are a clear example. In 2017, the MICROSCOPE mission could deliver the best test of the WEP^32^ thanks to the very quiet environment provided by the satellite surrounding the test masses of the differential accelerometer instrument. Atomic clocks and matter-wave interferometers reach their ultimate performance under free-fall conditions. In space, it is possible to reach interaction times between the atoms and the probing fields more than one order of magnitude longer than on the ground. In atomic fountain clocks, the stability is directly proportional to the interrogation time. This allows building instruments like the laser-cooled Cs clock PHARAO that, within a very small volume, mass and power consumption, can reach the same performance as atomic fountain clocks on the ground and even surpass them. PHARAO will soon fly to the International Space Station (ISS) as part of the ACES mission to test GR^33^. The benefits of cold atoms for acceleration measurements by matter wave interferometry are even higher, considering that the sensitivity of these instruments scales as the square of the interrogation time^34,35^.

The creation of BEC in space and first interferometric studies on a DLR-sounding rocket flight in 2017 marked the beginning of coherent atom optics and experiments with ultracold atoms in space^36^. Studying ultracold gases continued in orbit thanks to NASA’s Cold Atom Lab (CAL) operating since 2018 as a user facility. Only recently, in 2019, an advanced apparatus of CAL was followed to perform interferometric studies with BECs in orbit. CAL functionalities will be further extended by the DLR-NASA facility BECCAL. The rapid succession of new instruments shows the maturity of concepts to generate ultracold quantum gases with atom chips. Thanks to their modular designs extensions or adaptations of CAL and BECCAL are comparably fast and also of low costs. Next to studies of quantum gases at the lowest energies, they allow method development for quantum technologies and serve as pathfinder for more ambitious missions as STE-QUEST^37^.

Large velocity, velocity variations, and large variation of the gravitational potential are accessible on board a spacecraft, thus providing wide signals for testing GR and possibly detecting tiny violations of Einstein’s EP. As an example, precision measurements of the gravitational redshift require large variations of the gravitational potential that can only be achieved in space^33,38,39^. A variety of Standard Model Extension (SME) tests based on clock and atom-interferometry measurements are possible and have been proposed for space^40^.

Finally, the huge free propagation distances available in space call for tests with high-performance links, both quantum and classical, in the optical and microwave domains. Lunar laser ranging experiments continue challenging GR, in particular the Universality of Free Fall and the SEP^41^. High-performance radio link experiments have been designed to measure PPN parameters^42,43^. Quantum links exchanging entangled photons have recently been used to place boundaries on gravity-induced decoherence models^44^. Optical and microwave links are also providing access to networks of clocks both on the ground and in space to test GR and search for DM^33,38,39,45^.

## Ultracold atom physics in space

Nobel-prize-awarded landmark achievements such as laser cooling and BEC paved the way for cold-atom physics in space. Space experiments were pioneered by cold-atom clocks and, very recently, BECs were studied on a sounding rocket as well as on the ISS.

### BECs and quantum-degenerate mixtures

Due to the gravitational sag in harmonically trapped atoms or the limited free-evolution time, there are plenty of phenomena and unexplored regimes involving quantum-degenerate gases and mixtures whose investigation is impaired by the Earth’s gravity field. Magnetic levitation can be employed to compensate for the gravitational force^46^, but the technique has major limitations. Indeed, it cannot be applied to mixtures involving different internal states or multiple atomic species. Moreover, experiments exploiting Feshbach resonances to tune the inter-atomic interaction cannot be combined with magnetic levitation either. In all these cases the phenomena and regimes alluded to above are inaccessible to ground experiments and require microgravity platforms.

More specifically, the extended microgravity conditions afforded by space platforms such as the ISS offer unique opportunities in the following areas:

- *Scalar BECs:* Long free-evolution times for BECs with very low effective temperatures, gases with record-low entropy per particle, space atom laser, 3D bubble shells of trapped BECs.
- *Coherent atom optics:* Linear optics with nearly monochromatic matter waves, quantum reflection.
- *Spinor BECs and quantum gas mixtures:* Spinor BECs, Bose-Fermi mixtures, study of phase separation, quantum droplets (long-term dynamics in potential-free environment).
- *Strongly interacting gases and molecules:* Feshbach-molecule formation and Efimov physics.
- *Superfluid Fermi gases with tunable interaction*.
- *Critical phenomena near phase transitions*.
- *Entangled atoms*.
- *Quantum memories*.

BECs have already been created in space, both in sounding rockets^36^ and on the ISS^47^. Ultracold atomic bubbles have recently been observed in microgravity^48^. Furthermore, there are already plans for a second-generation device with many new capabilities to be operated on the ISS within a few years^49^. These experiments offer a large heritage for future space missions.

This toolbox can be exploited to include additional or other features bringing some of the novel experiments listed above in reach. Examples are the cooling of fermions in microgravity, dual-species experiments, or the study of critical phenomena near a phase transition. Pioneering work in this direction was the helium 4 specific heat measurement near the Lambda point realized on the ISS in 2003^50^. Such space experiment was the most precise test of universal exponents associated with a superfluid phase transition. With ultracold Bose and Fermi gases in microgravity, these critical exponents could also be tested in the strongly correlated regime.

The second class of applications is represented by quantum memories with extremely long-lived coherence time based on cold neutral atoms. In many ground experiments, the memory coherence time is limited by the confining potential that must compensate for gravity. In a micro-gravity platform with atom trapping at a magic wavelength, a memory coherence time over 10s seconds can be obtained.

Thirdly, high-end atom-interferometry methods can be implemented for testing the EP with unprecedented stringency. Beyond the development and the validation of new techniques for space-borne atom interferometry, such a device could be exploited for testing QM benefiting from the lowest energy scales nowadays accessible.

Moreover, there exist several concepts to establish entangled atoms at ultralow energy scales, based on the heritage of current space missions. Entangled atoms open up new avenues to test and explore quantum correlations and, hence, QM with massive particles. These sources allow addressing the quest of possible fundamental reasons fading these correlations over macroscopic times next to conventional technical reasons. In recent years, space-borne sources of entangled atoms came into reach as non-classical correlations could be demonstrated in ultracold atomic systems during free fall. Such experiments will take advantage of terrestrial microgravity platforms to be ready for space-borne experiments towards the end of this decade.

### Non-interferometric test of spontaneous wavefunction collapse

Ultracold atoms provide a powerful platform to test deviations from QM of the kind envisaged by wavefunction collapse models. The study of the expansion of a free non-interacting BEC already sets a competitive bound^51^ on the Continuous Spontaneous Localization (CSL) model^52^; this result was further improved using double-well systems^53^, but is still far from testing the entire parameter space of the model.

The experiment considered in^51^ was performed on Earth^54^, where a major obstacle was gravity, which limited the total duration of the experiment to about a few seconds. Recently, the proposal "CATinSpace: Cold Atoms Tests of the superposition principle in Space” was presented in response to the Call for Ideas to update ESA’s SciSpacE Physical Sciences roadmaps. There, it is suggested to test the CSL model with cold atoms on board the ISS, by monitoring the collapse-induced expansion of a BEC in a microgravity environment. A BEC with ideally 10^3^ or more atoms is prepared and cooled down with state-of-art techniques. Then the trap is released and the cloud is let free to evolve as long as possible; this is where the advantage of a microgravity environment enters. Last, the expansion of the cloud is measured, and compared with the CSL theoretical predictions, bounding in this way the collapse parameters. The great advantage of this approach is that it does not require to set the atoms in a quantum superposition.

Theoretical analysis^51^ shows that the size of the cloud grows with the cube of free-evolution time under the effect of the collapse noise, hence performing an experiment for longer times in a microgravity environment allows setting bounds, which are stronger than current ones. One can also consider a BEC with an attractive interaction: in such a case, the standard evolution predicts basically no expansion, so any increase of the position or momentum will be easier to detect. Preliminary calculation shows that in this way it will be possible to reach a sensitivity high enough to rule out the CSL model with the weak values for the parameters originally suggested by Ghirardi, Rimini and Weber^19^.

A detailed analysis of noise effects in a space environment for testing collapse models is under development. Contrary to a ground-based experiment, a space-based experiment can provide strong advantages in terms of external vibration, especially in the low-frequency regime. Several sources of vibrations are not present in space, as for example seismic ones. To be quantitative, the drop-tower in Bremen allows for up to around 9 s of free-fall, and is characterized by an acceleration noise of the order of $\sqrt{{S}_{aa}} \sim 1{0}^{-5}\,$m s${}^{-2}/\sqrt{\,{{\mbox{Hz}}}\,}$. This value can be compared to the impressive achievement of LISA Pathfinder of $\sqrt{{S}_{aa}} \sim 1{0}^{-15}\,$m s${}^{-2}/\sqrt{\,{{\mbox{Hz}}}\,}$, which already allowed to set a very strong bound on the CSL model for large values of *r*_C_.

## Atomic clock tests of GR

An important aspect of GR is that gravity affects time. Time flows differently in different gravitational potentials, an effect called gravitational time dilation or gravitational redshift.

Until 2018, the most precise measurement of the gravitational redshift was at 1.4 × 10^−4^ fractional inaccuracy level (1976 Gravity Probe A mission), realized by comparing two hydrogen masers at 1 × 10^−14^ frequency uncertainty level, where one maser was launched into space on a rocket to a maximum vertical height of 10,000 km, while the reference maser clock remained on Earth. This 45-year-old experiment was surpassed in 2018 by a detailed analysis of the clock signals on the two Galileo satellites which were inappropriately launched on an elliptical orbit. The redshift test was improved by 5.6 times, reaching 25 parts per million^38^. The need to improve on the above test result has motivated many researchers towards proposing new space missions. The Atomic Clock Ensemble in Space (ACES) mission will soon be flown on the ISS. Here, the microwave cold-atom clock PHARAO will deliver a signal with an inaccuracy of 1 × 10^−16^. Flying at an altitude of ≃400 km, the experiment will test the time dilation at the level of 2 × 10^−6^, providing a further improvement in sensitivity by one order of magnitude.

The developments in the field of atomic clocks in the last 10 years have opened up new exciting possibilities to test the foundation of GR. Indeed a new generation of atomic clocks has been established, the optical atomic clocks. They have been made possible by the development of lasers with superb spectral purity, of subtle atom manipulation techniques, and of the femtosecond frequency comb technique, which were awarded several Nobel prizes in recent decades. The potential of optical clocks relies on the access to narrow atomic transitions in the optical domain (*ν*_0_ ~ 10^15^ Hz) having a natural linewidth *δν*_0_ of a few mHz, corresponding to a transition quality factor *Q* = *ν*_0_/*δν*_0_ 5 orders of magnitude higher than achievable in microwave standards with *ν*_0_ = 10^10^ Hz and *Q* ~ 10^10^. In the last few years, this potential has been expressed, with groups demonstrating for the first time fractional stability and accuracy down to the 10^−18^ level or below^55^. This level is a factor of approximately 100 better than obtainable with the best microwave atomic clocks and current progress in this domain is rather fast.

Several national metrology institutes operate optical clocks in the 10^−18^ stability range, with strontium (Sr) lattice clocks, ytterbium (Yb) lattice clocks, and Yb^+^ or Al^+^ single ion clocks. Ground clocks may reach the 10^−20^ range in the next 5 years, thanks to a large number of quantum optics and laser specialists contributing to clock developments worldwide, especially in Europe, US, Japan, and China.

An impressive example of the performance of optical lattice clocks was recently given in Japan, where an optical lattice clock at RIKEN (Tokyo) was compared with a similar clock at the University of Tokyo, located at 15 km distance and linked by an optical fiber. The measured frequency difference was −709.5(28) mHz (corresponding to a relative uncertainty of 6.5 × 10^−18^). This is in agreement with the expected redshift of −707.48 mHz due to the gravitational potential difference over the 15 m height difference, which was independently measured by leveling techniques. This experiment already achieves an inaccuracy of 4 × 10^−3^ in testing the redshift. Considering that the height differences was really small, the achieved inaccuracy puts into evidence the tremendous potential of the optical clock technique, if the large height differences that space provides can be made use of. Strong progress is also occurring on implementing ultraprecise optical clocks capable of operating outside of the few advanced metrology laboratories. In Japan, one transportable optical clock was operated recently on the Tokyo Skytree radio tower and in Europe, three transportable optical lattice clocks have been developed, and one of them has already been transported between countries. The vision of availability, ten years from now, of a large set of optical clocks with 10^−19^ level performance that can be transported and operated anywhere on Earth is becoming realistic.

This progress has implications for space missions with optical clocks:

1. The mission will need to provide links capable of comparing ground clocks at the 10^−19^ level in a moderate integration time, the ground clocks being located anywhere on the Earth.
2. The number of ground clocks available for inter-comparisons will be large (>20).
3. The improving accuracy of ground clocks implies that more accurate tests of the gravitational redshift become possible when comparing ground clocks with space clocks.

### GR, time and frequency transfer, and geodesy

GR can be tested to high accuracy with a lattice optical clock in space and an optical time transfer link. Different scenarios allowing tests with increasing accuracy can be envisaged.

With an optical clock on the ISS:

- Test of the gravitational redshift in the Earth field with up to 100 times higher accuracy than ACES;
- Test of Local Position Invariance in the Earth’s gravitational field with up to 100 times higher accuracy than ground experiments;
- Test of the gravitational redshift in the Sun field with up to 10 times higher accuracy than with ACES;
- Worldwide comparison of ground optical clocks with applications to e.g. relativistic geodesy down to the 1 mm height resolution level (with improvements in modeling of relativistic frequency transfer and orbital motion);
- Search for DM or new physical fields that couple to ordinary matter leading to clock frequency variations of a different type;
- Dissemination of time and frequency worldwide, with 10^−18^ inaccuracy, on the time scale of a single pass of the ISS. Dissemination can be to ground, to satellites, or to tropospheric/stratospheric platforms.

With an optical clock on a highly elliptic orbit around Earth:

- Test of the gravitational redshift with up to 1000 times higher accuracy than ACES;
- Test of Local Position Invariance in the Earth’s gravitational field with up to 1000 times higher accuracy than ground experiments (with a two-species clock);
- Worldwide comparison of optical clocks, with applications to e.g., relativistic geodesy at the 1 mm level, thus supporting progress in optical clock development; dissemination of time worldwide to a vast range of users.

With an optical clock on an orbit to Mercury:

- Test of the gravitational redshift in the Sun’s gravitational field with up to 10^8^ times higher accuracy than previous space missions/solar spectroscopy;
- Test of Local Position Invariance in the Sun gravitational field with up to 100 times higher accuracy than ground experiments (with a two-species clock).
- Test of light propagation in the gravitational field (Shapiro time delay, light deflection).

A clock orbiting around the Earth and a space-to-ground link can be used to establish a network for the comparison of atomic frequency standards, both space-to-ground and ground-to-ground, on a worldwide scale. Ground clocks are today compared via the Global Navigation Satellite System (GNSS) or Two-Way Satellite Time and Frequency Transfer (TWSTFT). IPPP (Integer Precise Point Positioning) processing of GPS data can provide clock comparisons at the 1 × 10^−16^ level after less than one week of integration time^56^. However, the high stability and accuracy demonstrated by optical clocks are now demanding a clock comparison infrastructure at least two orders of magnitude beyond current operational systems. The ACES mission will help bridge this gap by providing means for comparing clocks on the ground to 1 × 10^−17^ after a few days of integration time. The next generation of time and frequency transfer systems is expected to reach the 1 × 10^−19^ uncertainty level in the same measurement duration. Coherent optical links in free space or through optical fibers have already demonstrated such performance^57,58^. Upgraded versions of the ACES microwave link are currently under development. A fiber link network for comparing distant clocks is already connecting European metrology institutes (SYRTE to PTB, SYRTE to NPL, PTB to MPQ, SYRTE to INRIM, and INRIM to LENS) and additional links will become available in the coming years. This infrastructure, distributing time and frequency on a regional scale, will be the natural completion of a space-based system connecting clocks over intercontinental distances and in a worldwide network.

### Lorentz symmetry tests and CPT violations

State-of-the-art single ion clocks at the 10^−18^ level were recently demonstrated to be able to test local Lorentz invariance^59^ through sidereal modulations of the frequency offset that hypothetical violations of Lorentz invariance would cause. In fact, already in this case, the observed absence of such modulations was used to deduce stringent limits on Lorentz symmetry violation parameters for electrons in the range of 10^−21^, improving previous limits by two orders of magnitude.

Moreover, being a consequence of Lorentz symmetry, CPT-symmetry is a likewise fundamental property of all our theories, a violation of which would imply Lorentz symmetry violation. Such a violation of CPT-symmetry would show up in unequal moduli for the *g*-factors of the proton and anti-proton, which were, e.g., constrained to below 1.5 parts per billion by means of a two-particle spectroscopy method in a cryogenic multi-Penning trap^60^. Lorentz- and CPT-violating terms of the non-minimal SME can also be constrained by searches for asymmetries in the dark-matter interactions of protons and antiprotons^61^.

## Matter-wave interferometry tests of GR and QM

### Atom interferometry

Atom interferometers^62–64^ can address central questions in QM and GR. Recent research has shed new light on traditional concepts of GR in the context of matter-wave interferometry showing that the latter enables tests that are rather complementary to classical ones regarding the universal free fall of matter as well as the UGR. Moreover, the progress in quantum engineering of matter waves rises prospects for performing tests of unprecedented rigor for the Eötvös ratio in the 10^−17^ range.

On ground, atom interferometers have already been successfully exploited for high-precision measurements in fundamental physics and for practical applications^65^. However, thanks to the extended microgravity conditions afforded, it is in space that their full potential can be unleashed and where unprecedented sensitivities could be attained.

Only recently, a comparison of matter wave interferometers based on rubidium isotopes reached a sensitivity of 10^−12^ ^66^ leaving still quite a gap to the performance anticipated for space-borne tests on a dedicated satellite. Perspectives for bridging the remaining gap and for reaching sensitivities beyond current results are opened up by elevator tests allowing for better statistics and a better microgravity environment than other platforms.

Having pioneered dual species interferometry based on potassium and rubidium both on ground^67^ and during parabolic flights^68^, Europe has a considerable heritage in performing such tests. Moreover, several sounding rocket missions carrying a dual-species interferometer to space are already foreseen for the next years. Hence, within this decade, one can expect terrestrial microgravity experiments to improve state-of-the-art quantum tests by about two orders of magnitude and rising the TRL for space-borne tests.

#### WEP tests

Concepts for quantum tests of the EP have been already established both for the ISS as well as for satellites. A satellite-based quantum test of the WEP is pursued by a large European consortium^34^. Considered as not mature enough for the ESA’s Cosmic Vision program, it is now proposed for Voyage 2050 acknowledging the recent progress in the field^69^. The ISS is not the ideal platform for WEP tests, but it can still provide a competitive accuracy in the determination of the Eötvös parameter thanks to the high level of control that an atom-interferometry measurement can offer on vibrations and rotations^70^.

It has been emphasized that the co-location of the different atomic species in tests of UFF based on atom interferometry, which is an important contribution to systematic effects, poses a major challenge in order to achieve such high sensitivities. Indeed, target sensitivities at the 10^−17^ level imply very stringent requirements on the initial kinematics of the two atomic clouds: their relative initial position and velocity need to be controlled at the level of a few tens of pm and pm/s respectively. This is technically very demanding and, moreover, its verification under the same experimental conditions would require a large fraction of the mission lifetime. Fortunately, an effective mitigation technique has recently been proposed: by suitably adjusting the frequencies of some of the laser pulses, it is possible to compensate for the effects of gravity gradients and relax the requirements on the initial kinematics by several orders of magnitude^71^.

The experimental implementation of the gravity-gradient compensation technique has been successfully demonstrated in ground experiments^72,73^, and it is an important element of a recent atom-interferometric test of UFF at the 10^−12^ level^66^ with prospects for further improvement in the near future. Furthermore, in space missions, it can be combined with orbital demodulation methods, so that sensitivities up to 10^−18^ can be reached with moderate requirements on the initial co-location^74^.

On the other hand, the progress made in performing experiments with BECs in space^36,47^ offers also new prospects to exploit the heritage for a quantum test in orbit, on the ISS. Albeit offering a lower performance than satellite missions, experiments on the ISS are anticipated to reach an intermediate level of stringency between 10^−13^ and 10^−16^ in the Eötvös ratio, depending on the experimental design^70,75^. As initial steps in this direction, there are already preliminary atom-interferometry experiments underway with the CAL on the ISS performed in collaboration with NASA, and more advanced ones will be possible thanks to the next-generation device BECCAL^49^, which will feature extended atom-interferometry capabilities.

#### Dark energy

As discussed in “Open problems in fundamental physics”, atom interferometers can be much more sensitive to forces mediated by chameleon and the symmetron fields^15^ and have already been exploited to exclude part of the parameter space for such kinds of models^76–78^. However, further constraining these models will require longer interferometer times where the atoms spend a large fraction of the interferometer time close to the source mass and this can be naturally accomplished in microgravity^16,79^.

#### Dark matter

Pairs of atom interferometers in space separated by long baselines (from thousands to millions of km or more^80^) and interrogated by common laser beams propagating along that baseline can be exploited to search for DM candidates corresponding to ultralight scalar fields^14^. In order to avoid otherwise extremely demanding requirements on laser phase stability, a new kind of atom interferometer based on single-photon diffraction needs to be employed^81^.

The available platforms and resources do not fulfill the requirements for a fully-fledged mission^35,82,83^. However, demonstrators involving single-atom interferometers or even pairs of interferometers separated by small distances could help to boost the required technology readiness for future dedicated missions. Since they employ the same kind of atoms (e.g., Sr or Yb) and lasers (laser cooling and clock transition) as optical atomic clocks, joint efforts with plans for an optical clock in space (I-SOC)^84^ should be possible.

Furthermore, by making use of simultaneous pairs of laser pulses driving the clock transition that can simultaneously diffract the two internal states^85^, it would be possible to perform WEP tests with atoms in a quantum superposition of internal states (in this case the two clock states, with an energy difference of the order of a few eV). Compared to previous ground experiments with superpositions of hyperfine states^86^, this would enable longer interferometer times (and hence higher sensitivity) and would increase the energy difference between the two internal states involved by five orders of magnitude.

#### Lorentz symmetry and CPT violations

Up to now, only about half of the coefficients for Lorentz violation, in the context of the fermionic sector of the minimal SME in Minkowski space-time, have been investigated experimentally. However, some of these open parameters can be constrained in the future by considering gravitational couplings in the fermionic sector of the SME, with a particular interest in the coefficients for baryons and charged leptons, in principle unobservable in Minkowski space-time, which could be largely due to gravitational countershading.

A major class of experiments that can achieve sensitivity to these coefficients involves tests with the ordinary neutral matter. They are analyzed via a Langrangian describing the dynamics of a test body moving near the surface of the Earth in the presence of Lorentz violation, revealing that the gravitational force acquires tiny corrections both along and perpendicular to the usual free-fall trajectory near the surface of the Earth, and the effective inertial mass of a test body becomes a direction-dependent quantity. The tests can be classified as either gravimeter or WEP experiments and as either force-comparison or free-fall experiments.

Atom interferometry provides extremely sensitive and accurate tools for the measurement of inertial forces and is then of particular interest to test Lorentz violations. During the free fall of cold atoms, they experience a sequence of three laser pulses, that split and recombine the atomic wavepackets. Operation of atom interferometers in microgravity is expected to increase the duration of free-fall and then to enhance the performance of such sensors. Consequently, we expect to increase their sensibility to possible Lorentz violation in the gravity-matter couplings of SME.

### Large-mass interferometry

Beside the mature science and technology of cold atoms, there is a growing number of large-mass experiments to test various aspects of fundamental physics, ranging from testing the quantum superposition principle, gravitational decoherence, relativity, and gravitational waves, the interplay between QM and gravity as well as predictions for DM and DE. Experiments with large-mass systems (typically from 10^9^ to 10^15^ atoms) include non-interferometric opto/electro/magneto-mechanical systems^87^ as well as matter-wave interferometric experiments with molecules and nanoparticles^88^. Large-mass systems push the envelop of realization of quantum states towards the macroscopic domain, while providing an ultra-sensitive test bed for the standard model and exotic forces and acceleration. Similarity with cold atomic manipulation is the goal to quantum control the center of mass motion and most of the ideas for the fundamental test with atoms can be translated forward to heavier particles.

At the same time, there is an important development going on in our approach to fundamental physics by challenging our common understanding of nature, while taking a fresh view on the topics of relativity and QM. Some ideas have been put forward and have to be evaluated by the scientific community in the context of the best choice for an experimental platform in order to test them. In this respect, these new ideas are less mature, when compared to some of the other ideas discussed in this paper, but there is for sure a new horizon.

All the experiments discussed here will benefit from extended periods of free evolution in the micro-gravity environment available in space.

#### Non-interferometric tests with large-mass systems

Proposals are based on the mature experiments of optomechanics and especially levitated optomechanics and include testing of predictions from GR such as gravitational waves in a higher frequency domain complementary to large-footprint experiments such as LIGO, VIRGO, and GEO600 on compact designs and geometries^89,90^, and the testing of classical gravity and space-time curvature^91^, while pilot experiments on Earth have been realized already^92^. Using large-mass systems to probe the high-energy particle physics sector beyond the standard model includes testing DM candidates^93,94^ as well as DE^95^. Experimental geometries for gravitationally interacting one- and two-mass systems include ideas for testing the gravitational field generated by a massive quantum system^96,97^, but also include probing the GR frame dragging effects^98^. Last but not least, the quantum superposition principle has been tested already non-interferometrically in the lab^30^, but would certainly benefit from the envisaged space environment as well.

#### Interferometric tests with large-mass particle systems

Large-mass matter-wave interferometers in space will be able to test DM candidates^93,99–101^, as well as quantum superpositions in the large-mass limit of macroscopicity, which has been put forward as the MAQRO proposal^102^. The idea has been successfully evaluated within the Quantum Physics Platform (QPPF) CDF study by ESA already and is awaiting technology development of components, which is underway in the optomechanics community^103^. The design of a matter-wave interferometer for nanoparticles fit for space have been theoretically proposed and discussed^104–106^ using different types of coherent beam splitters for nanoparticle matter-waves. A progressive idea is to utilize the rotational degree of freedom of large-mass systems, actually in interferometric and non-interferometric settings, to test QM in the macroscopic domain^107,108^. Again, key to conduct those experiments is access to extended periods of time in micro-gravity environments. A review summarizing the experimental challenges of interferometric experiments with large-mass particles has recently appeared^109^.

#### Testing the interplay between gravity and QM

Proposals have been worked out in the context of gravitational decoherence and semi-classical gravity^110,111^, the role of gravity in the collapse of the wavefunction according to the Diosi-Penrose ideas^21,112–114^ as well as in the context of stochastic gravity^115–118^. Ideas that attracted much attention include a new take on testing quantum gravity by using quantum information protocols for the state preparation of large-mass systems^119–122^. Further ideas have been put forward to test gravitational decoherence and general relativistic time dilation effects also with large-mass systems in interferometric settings^123^ and a scientific debate is underway to explore the correct physics description and solid prediction of the effects^85,124,125^. Furthermore, experiments to test QM in accelerated reference frames, aiming to exploit the correspondence between acceleration and gravity utilized by the EP and the use of stark accelerated systems. First experiments have been performed in research laboratories and with quantum states of light, such as entangled states^126^ or those showing other strong and non-classical correlations^127^, but can also be extended to large-mass systems and indeed the space settings^128^.

Probably the most influential proposal for testing the overlap between QM and gravity is about an indirect test of gravity to act as a quantum channel between two massive quantum systems and for the generation of quantum entanglement between them^129,130^. While these proposals triggered a very fruitful discussion about what such an experiment would actually prove, it becomes apparent that also (technically maybe simpler) single-mass quantum superpositions and gravity-induced collapse of their massive wavefunction would allow for such a test. What is common to all those ideas is that they aim to test the interplay between QM and gravity (or GR) in a new regime, namely the low-energy (non-relativistic) regime, which is very different from the traditional high-energy (highly relativistic) regime of the graviton. While it is clear that testing for high-energy predictions of quantum gravity will need very futuristic particle accelerators of practically impossible dimensions, the new low-energy regime allows for a completely different class of experimental tests based on optomechanics and spin physics. Questions like how the gravitational field of a spatial quantum superimposed single mass actually looks like are central to this new approach.

## Classical and quantum links

The exchange of photons between Earth stations, spacecraft, or laser retroreflectors has been the primary tool to test gravity in the solar system. While in the past most of the observable quantities (travel times of photons and their time variation, VLBI) have been provided using microwave links, in the next future it is conceivable to see an increasing role of laser links. An important, past example has been the Lunar Laser Ranging program, which has provided important constraints on PPN parameters as well crucial information on the interior structure of the Moon. In the future, we will see substantial improvements in both techniques. Microwave links have been demonstrated to deliver round-trip light-time measurements corresponding to distances of 1 cm over distances of 1 AU. Laser links are evolving towards coherency (with the use of laser transponders), offering far better accuracies than laser bouncing by means of retroreflectors.

Optical links between ground stations on Earth and orbiting satellites, or arrays of linked satellites, also open the way to a number of fundamental physics experiments involving the exchange of quantum states. Such experiments may explore the interplay between QM and GR by studying quantum photonic entanglement in curved space-time, lead to more precise measurements of physical constants and time dilation by synchronizing terrestrial and orbital atomic clocks, and enable tests of DM or exotic light fields. We expect that the technology towards these goals will evolve considerably within the next decade, driven also by their applications to space-based quantum communication and to better navigation for spacecraft. We note that the technical challenges of establishing an optical link (classical or quantum) are nearly identical. Quantum links must often deal with lower power levels (e.g., single-photon states) and may often require more precise control over the spatial, temporal, and frequency modes of the light signals.

### Solar system tests with classical links

The solar system continues to be a valuable laboratory for tests of gravitational theories in the weak field limit. Its main advantage is that all measurements are carried out in a well-known and controlled environment. Strong field tests made possible by current and future gravitational wave detectors, besides testing different aspects of gravity, cannot claim the same precise knowledge of the dynamical environment. Solar system tests rely almost entirely on the exchange of photons between Earth and a distant spacecraft. At the moment deep space links are established using microwave frequencies, in particular Ka-band (32–34 GHz) for higher measurement accuracy. In the future laser, links may offer improved accuracies and a more accurate metrology system.

There are laser retroreflector arrays (LRAs) on the Moon for ~50 years, deployed by Apollo 11, 14, and 15 astronauts^131^ and by the Lunokhod 1 and 2 rovers. There were no laser retroreflectors on Mars, until a downsized, lightweight LRA (or “microreflector”)^132,133^ was deployed on Mars with NASA’s InSight lander in 2018^134^. Apollo and Lunokhod LRAs are positioned by time-of-flight measurements of short laser pulses shot by ground stations of the International Laser Ranging Service (ILRS, ilrs.gsfc.nasa.gov). This is the so-called Lunar Laser Ranging (LLR) geodetic technique, which is performed regularly by three ILRS stations: Grasse in France (in service for the longest time), APOLLO (Apache Point Lunar Laser-ranging Operation) in the USA (the most modern and accurate) and MLRO (Matera Laser Ranging Observatory) in Italy. Several other ILRS stations are starting or testing LLR, in China, Europe, and Russia. Microreflectors are designed to be positioned by orbiting spacecraft equipped (for example) with laser altimeters like NASA’s Lunar Reconnaissance Orbiter (LRO, currently active) and NASA’s Mars Global Surveyor (MGS, active until 2007). This is an “inverse” Satellite Laser Ranging (SLR) geodetic measurement if compared to the routine operation of the ILRS (laser stations on the ground and LRAs on orbiting satellites).

Already in 2005 laser links at 1064 nm have been proven^135^ between ILRS stations (including the NASA-GSFC 1.2 m laser telescope) with: (1) MOLA (Mars Orbiter Laser Altimeter) onboard MGS at distances of 80–100 million km: (2) MLA (Mercury Laser Altimeter) onboard MESSENGER (MErcury Surface, Space ENvironment, Geochemistry, and Ranging) at a distance of about 25 million km. The former was a laser uplink (Earth to spacecraft), while the latter was the first uplink and downlink laser communication at interplanetary distances. In the 2010 decade, laser uplink campaigns were performed at 532 nm from multiple ILRS stations to LOLA (Lunar Orbiter Laser Altimeter) onboard LRO^136^. In 2013 successful high-rate lasercom to and from Moon orbits (uplink and downlink at 1550 nm) was demonstrated by the LLCD payload (Lunar Laser Communications Demo) onboard NASA’s LADEE orbiter (Lunar Atmosphere and Dust Environment Exploration)^137^. Also, ESA’s Optical Ground Station (OGS) in the Canary islands participated in this international lasercom campaign. Finally, in 2018 and 2019 the Grasse ILRS station was also able to perform SLR at 1064 nm to an LRA onboard the anti-nadir side of LRO^138^.

#### Radio links

Solar system tests with radio links rely essentially on two methods:

1. The measurement of the time delay, frequency shift, and angular deflection of radio beams (in the latter case using extragalactic sources, not spacecraft);
2. The precise monitoring of the motion of solar system bodies is carried out using active spacecraft tracking.

These tests are enabled by precise measurements of spacecraft range and range rate. Several technological developments have been carried out in the last decade in order to improve measurement quality. The most important are listed below:

1. Use of higher frequency or multi-frequency radio links to reduce or suppress charged particle noise;
2. Use of more precise ranging systems by means of pseudo-noise modulation codes at higher chip rate;
3. Use multistation tracking, with a small listen-only antenna located at a high altitude) to reduce tropospheric and mechanical noise in Doppler measurements.
4. Use stable clocks onboard a spacecraft to establish precise one-way radio links for Doppler measurements.

It is important to point out that in order to fully exploit improved measurement systems, a corresponding improvement of the dynamical model of the spacecraft and the solar system itself is necessary. On the spacecraft side, the measurement of non-gravitational accelerations, or even the transition to drag-free systems, is a necessary step. Accelerometers are the simpler solution, but the real difficulty, requiring considerable technological development, is the extension of the operational band to lower frequencies (10^−7^−10^−6^ Hz).

After the start of operations of GAIA, the launch of BepiColombo is probably the most relevant event for solar system missions with a substantial set of objectives in fundamental physics. BepiColombo uses a multilink radio system for full plasma calibration both for Doppler and range measurements, and 24 Mcps pseudo-noise range modulation (corresponding to a wavelength of 25 m). Early results from inflight tests show an accuracy of the ranging system at the level of 1-2 cm over 4 s integration time, for the entire duration of a pass (about 8 h). Ground and onboard delay calibrations were crucial to attaining such a unique result for a radio system. If this measurement accuracy will be demonstrated to be an absolute one (i.e., an absolute round-trip light-time measurement), it will be possible to resolve the phase ambiguity, at least for the X band signal.

BepiColombo will exploit six solar conjunctions during a cruise to carry out the classical test of time delay and frequency shift, with the prospect of significantly improving the Cassini result for the PPN parameter *γ* [(2.3 ± 2.1) × 10^−5^]. During the orbital phase, BepiColombo data, combined with solar system dynamics knowledge acquired by other missions (past, ongoing, and close to launch) is expected to improve significantly the accuracy from almost all PPN parameters. Table 1 summarizes the expected accuracy under the assumption of 20 cm ranging accuracy (according to instrument specifications) rather than actual performance (2 cm) and different assumptions for the analysis.

**Table 1:** Tests of gravitational physics with radio links.

Parameter	Imperi et al.	De Marchi and Cascioli
*γ*	6.6 × 10^−7^	1 × 10^−6^
*β*	4.5 × 10^−7^	1.7 × 10^−5^
*J*_2_	1.37 × 10^−9^	2.8 × 10^−9^
*η*	1.36 × 10^−6^	6.9 × 10^−5^
*α*_1_	1.2 × 10^−7^	3.4 × 10^−7^
*α*_2_	4.6 × 10^−8^	6.7 × 10^−8^
*GM*_⊙_ (km^3^ s^−2^)	0.015	0.08
*ζ* (yr^−1^)	3.2 × 10^−15^	9.2 × 10^−15^
*λ*_*g*_ (km)	−	<1.1 × 10^14^

Expected accuracies in PPN parameters, gravitational parameter of the sun, relative time derivative of the Newtonian gravitational constant (*ζ*), and Compton wavelength of the graviton (*λ*_*g*_), using 20 cm range accuracy (instrument requirement) and different assumptions in the analysis, and for a 2-year mission duration (from ref. ^185^).

GAIA is also expected to release soon a substantially improved measurement of *γ*, using astrometric measurements. The diversification of methods in precise tests of GR is of course of the utmost importance.

Being carried out in a fully relativistic frame, the generation of solar system ephemerides offers another method to test gravity laws (see^139^, with earlier references therein). The BepiColombo data are expected to improve significantly our knowledge of solar system dynamics.

Among future projects being proposed to improve solar system ephemerides, the TRILOGY concept^140^ is especially interesting. The main goal of TRILOGY is twofold: on one hand, to improve the range measurements by using interplanetary laser links; on the other hand, to remove degeneracies in the orbital solutions related to the fact that all measurements are carried out from the Earth. Using planetary orbiters (or even landers) exchanging laser pulses would provide a more robust determination of the planetary ephemerides and the associated relativistic parameters. In addition, TRILOGY could measure the expansion of the solar system ensuring the mass loss from the sun.

The TRILOGY concept would certainly require significant technological development in interplanetary laser links and accelerometers. It will certainly benefit from the technological fallout from LISA and the space gravitational wave detectors proposed for the very low-frequency band (roughly 10^−4^ − 1 Hz).

#### Laser links

For about 50 years LLR to Apollo/Lunokhod Cube Corner laser Retroreflector (CCR) arrays supplied accurate tests of GR and new gravitational physics: possible changes of the gravitational constant $\dot{G}/G$, weak and SEP, gravitational self-energy (Parametrized Post Newtonian parameter *β*), geodetic precession, inverse-square force-law^141–143^, space-time torsion^144,145^ and nonminimally coupled gravity^146,147^. LLR has also provided significant information on the composition of the deep interior of the Moon, complementary to that of NASA’s mission GRAIL (Gravity Recovery And Lunar Interior Laboratory). In fact, already in the later 1990s LLR first provided evidence of the existence of a fluid component of the deep lunar interior^148^, confirmed later by a re-analysis of Apollo lunar seismometry data in 2011^149^. Therefore, Apollo/Lunokhod LRAs have supplied the first realization of a passive Lunar Geophysical Network (LGN) not only for precision tests of GR but also for lunar planetary science^150^. However, nowadays they only allow slow statistical improvements with data accumulation, which does not support the priorities of the modern science program.

For Moon missions, the relevant laser ranging instruments are full-size LRAs for direct LLR from Earth. In 1969 multi-CCR arrays contributed a negligible fraction of the LLR error budget. Since laser station range accuracy improved by more than a factor 100, now, because of lunar librations, the Apollo/Lunokhod LRAs dominate the LLR error budget due to their multi-CCR geometry and large geometric size. For direct LLR by ILRS, a next-generation, single, large CCR payload has been developed by a US-European collaboration, which is unaffected by lunar librations, that supports an improvement of factor 100 of the space segment contribution to the LLR error budget (see, refs. ^142,143,151^). This instrument has a mass of the order of kg.

Any next-gen retroreflector will improve the fundamental physics (and geophysics) reach over Apollo/Lunokhod. The expected improvements of fundamental tests of gravity with three or more next-gen retroreflectors compared to Apollo/Lunokhod LRAs and as a functions of the LLR error budget are reported in Table 2. This analysis^142^ is performed with the Planetary Ephemeris Program (PEP) developed by I. Shapiro et al. (PEP is described for example in^152^). The test of the inverse-square force-law (1/*r*^2^) reported at the last row of Table 2 refers to the test of additional Yukawa-like potential, with a standard parametrization in terms of a range *λ* (at the exponent) and a multiplicative strength *α*. LLR probes the Earth–Moon distance, which is *λ* ~ 384,000 km.

**Table 2:** Tests of gravitational physics with next-generation lunar laser retroreflectors.

Gravitational	Apollo/Lunokhod	Next generation	Time	Ultimate goal
measurement	LLR accuracy	LLR accuracy	scale	LLR accuracy
	(~few cm)	(~1 mm)		(~0.1 mm)
WEP	$\left|\frac{{{\Delta }}a}{a}\right|\,<\,1.4\times 1{0}^{-13}$	10^−14^	Few years	10^−15^
SEP	$\left|\eta \right|\,<\,4.4\times 1{0}^{-4}$	3 × 10^−5^	Few years	3 × 10^−6^
*β*	$\left|\beta -1\right|\,<\,1.1\times 1{0}^{-4}$	10^−5^	Few years	10^−6^
$\frac{\dot{G}}{G}$	$\left|\frac{\dot{G}}{G}\right|\,<\, 9\times 1{0}^{-13}{\rm{yr}}^{-1}$	5 × 10^−14^	~5 years	5 × 10^−15^
Geodetic	6.4 × 10^−3^	6.4 × 10^−4^	Few years	6.4 × 10^−5^
precession				
1/*r*^2^ deviation	$\left|\alpha \right|\,<\,3\times 1{0}^{-11}$	10^−12^	~10 years	10^−13^

This specific compilation reports: tests of gravitational physics (1st column) performed with current LRAs and associated LLR error budget (2nd column^141^); test improvements with current LRAs plus next-gen retroreflectors and associated improved error budget (3rd column) expected in approximate, reference periods (specified at the 4th column)^142^; the ultimate LLR goal in terms of test accuracies and LLR error budget supported by next-gen retroreflectors (5th column), to be reached with multiple lunar missions (NASA-Artemis^186^, ESA-E3P^187,188^, other national/international programs), as well as progressive improvements of lunar orbit software (like PEP^142^ reported here and other ephemerides software systematically reviewed by Fienga et al.^139^).

For Mars missions, the relevant instruments are microreflectors with masses of the order of a few tens of grams that are positioned by laser ranging from Mars orbiters. Direct laser ranging from Earth-like for the Moon is not practically feasible. ESA’s ExoMars Schiaparelli, which unfortunately failed its landing in 2016, was carrying a microreflector^153^ like the one on InSight. Two additional micro reflectors will be deployed on the surface by NASA’s Perseverance and ESA’s ExoMars rover missions in 2021 and 2023, respectively^154^. Similar instruments can be proposed for the Mars Sample Return program of NASA and ESA: one for ESA’s Sample Fetch Rover and one for NASA’s Sample Retrieval Lander^155,156^.

To address the fundamental physics reach with Mars surface laser retroreflectors, we performed simulations of the contribution of a five-microreflector from the Mars Geophysical Network (MGN,^157^) to test GR by means of the PEP software. Under specific and conservative assumptions (described below) the contribution of this MGN is found to improve the measurements of $\dot{G}/G$ and of *β* (see Table 3). *γ* is used as a control observable, by comparing its estimate with measurements by Cassini and the ESA missions BepiColombo and GAIA (Global Astrometric Interferometer for Astrophysics)^158^.

**Table 3:** Tests of gravitational physics with first-ever martian laser retroreflectors.

Time/*σ*(CCR)	∣*β* − 1∣ accuracy	∣*γ* − 1∣ accuracy	$\dot{G}/G$ accuracy
10 years/10 m	1.5 × 10^−4^	7.0 × 10^−4^	3.5 × 10^−14^
10 years/1 m	3.4 × 10^−5^	1.4 × 10^−5^	1.1 × 10^−14^
10 years/10 cm	7.1 × 10^−7^	3.0 × 10^−6^	2.6 × 10^−15^
Accuracy	<1 × 10^−4^	2.3 × 10^−5^	9 × 10^−13^
With data/mission	LLR, MESSENGER	Cassini	LLR

The results presented here are obtained with a five-retroreflector MGN (PEP simulations).

Table 3 is obtained under the following assumptions:

- Hypothetical MGN with coordinates: Phoenix (68N, 234E), Viking 1 (22N, 50W), Viking 2 (48N, 258W), Curiosity roving region (4S, 137E), Opportunity roving region (2S, 354E). This is a non-ideal MGN, since almost all nodes are in the northern hemisphere.
- One laser orbiter observation every 7 Sols. This takes into account weather conditions, although for example, the visibility of Curiosity from MRO (Mars Reconnaissance Orbiter) is about once/Sol (source: NASA).
- *σ*(*CCR*) is the positioning accuracy of the MGN node (the microreflector) on Mars. *σ*(*CCR*) = 10 m and 1 m can be obtained by adding the Earth-Mars orbiter positioning by radio science and the orbiter-reflector positioning by laser ranging/altimetry. This would give significant improvements, since the current accuracy of Mars ephemeris is 50-100 m (see^153^ for a discussion). To reach *σ*(*CCR*) ~ 10 cm, future Earth-Mars orbiter optical links would be required.

### Fundamental physics with entangled photons

Ground-to-space optical links provide an unparalleled experimental framework for testing phenomena arising from the interplay of GR with QM. Such links offer experimental conditions impossible to achieve on the ground, in terms of gravitational potential variations, length scales, and relative velocities, that are crucial for verifying these phenomena. A scenario of particular interest regarding gravitational decoherence, currently studied in the framework of the Space QUEST mission^159^, concerns the observation of the strength of quantum correlations using photonic states. In this scenario, a quantum mechanical system consists of entangled-photon pairs; one photon of each pair is detected on the ground while the other travels uplink to the ISS. Similar experiments involving long-baseline quantum links between ground stations and the Lunar Gateway have been proposed in the context of the DSQL mission^160^.

Interestingly, there is a number of theoretical models attempting to predict how quantum correlations would evolve in the presence of such a curved space-time, with contradicting results. Standard QM predicts no additional decoherence due to the difference in gravitational curvature between the two-photon paths in such an experiment. Other theories, however, predict various types of effects. More specifically, the event operator formalism studied in Space QUEST^161,162^ predicts a gravitational decoherence effect due to a speculative nonlinear back-action of the metric on the quantum fields that leads to particle loss into a causally disconnected region of space-time. Furthermore, this type of decoherence effect is expected to be seen only by entangled systems, which means that purely classical correlations would not be affected. The prospect of bringing experimental evidence to this discussion opens up exciting possibilities for testing QM and GR together using technology conceivable within the next few years. Preliminary tests, with negative results, have already been conducted with the Chinese satellite Micius^44^.

Exchanging quantum states not only results in entanglement distribution but can be used for pico second-level clock synchronization which is crucial for navigation. By linking orbital and terrestrial atomic clocks, we can potentially measure changes to physical constants and make more precise measurements of time dilation. In ref. ^163^ the authors propose a way to measure time dilation when a single photon follows multiple paths in a Mach-Zehnder interferometer; see also ref. ^160^. This proposal is based on an optical analog of the Collela, Overhauser, and Werner (COW) experiment, which tested gravitational effects on matter interferometry in the Newtonian regime^164^. Similar experiments with processing polarization states to serve as a local clock^165^ and others using a "folded” interferometer with a single Earth orbiter and a ground station^166^ have also been proposed. With an array of synchronized atomic clocks all exchanging quantum states, the state of an atom in one clock can be teleported to another for high-precision comparative measurements. A large in-orbit array of synchronized clocks can be used to test for topological defects which could be a consequence of DM^167^.

Some modified theories of gravity predict the existence of some sort of screening scalar fields, such as Chameleon fields^168^. Using the phase picked up by the propagation of photons between Earth and a satellite link^169^, it may be possible to search for such Chameleon fields^170^. To some extent, the desired types of phase shifts were already observed in the COW experiment^164^. Further, while creating large baseline single-photon interferometers may seem daunting, experiments like ref. ^171^ have already demonstrated interference from time-bin entangled photons after traveling for ≈5000 km in free space.

The entanglement of massless fields in curved space-time predicts several potential experiments where the nature of entanglement is changed by the presence of gravity. Reference ^170^ offers a review of several such effects. For example, curved space-time affects the entanglement of Gaussian states^172,173^, two mode-squeezed states^174^, and multipartite W-states^175^.

Using a network of quantum sensors in orbit can be extremely useful to measure phenomena like Exotic Light Fields^176^ (which are predicted in several Beyond the Standard Model (BSM) theories^177^).

The gravitational decoherence phenomenon on the entangled photons, if present, would be weak for quantum signals sent to the low-Earth-orbit ISS. Dispersion of the quantum signals imposed by air could also pose a crucial obstacle to these measurements, as it could potentially cover up the effect. It is expected however that the effect would be measurable and an attempt to measure it is crucial to verify the standard theories. Since this would be the first experiment of its kind to test QM in a changing gravitational field, it would ideally allow us to place a bound on the maximum possible de-correlation due to gravity. This would help differentiate between different classes of theories pertaining to GR as well as QM, and to narrow down the approaches that have been put forward to describe the precise mechanism of quantum decoherence and its relation to gravity. Of particular interest from a fundamental point of view are radical objective state-reduction models that call for a breakdown of QM^178,179^. These are based on a thought experiment of Penrose, in which it was argued that a massive object placed in a superposition should quickly decohere in the position basis due to the inherent uncertainty induced in the space-time metric. By contract, the event operator formalism motivating the Space QUEST experiments is based on a thought experiment on the self-consistent dynamics of quantum systems near closed time-like curves due to Deutsch^180^. It considers exotic space times in which gravity creates closed time-like curves and hence permits time travel into the past. Deutsch argued that the usual paradoxes associated with such solutions of GR can be resolved by QM. He does not attempt to quantize gravity, but considers quantum systems localized to semi-classical trajectories in a classical background space-time, and argues that a system scattering from a closed time-like curve in space-time exhibits globally nonlinear and non-unitary dynamics. The event formalism extrapolates Deutsch’s model to massless fields propagating in a globally hyperbolic space-time background, in which case it predicts a decorrelation of entanglement due to gravitational curvature^161^. Unlike Penrose and other models that also treat space-time classically and posit a non-linear dynamical equation, the event formalism has a number of novel features: it predicts decoherence only for entangled systems and not for single systems in a superposition; the effect is in principle reversible by further gravitational interactions (hence it is better called ‘decorrelation’ than decoherence); and it may exhibit information processing power greater than that of standard QM^181^.

In addition to testing the above theories, ground-to-space optical links and the advanced quantum (and associated classical) technologies developed in the framework of the Space QUEST mission (entangled-photon source, single-photon detectors with low timing jitter and dark counts, clock synchronization techniques) can be used for fundamental tests relevant for quantum information^182^. In particular, it is unknown to date at which length scale the violation of Bell inequalities as a signature of the non-locality of QM may still be confirmed or whether such fundamental features of QM break down. Beyond their foundational interest, such tests are crucial for validating the concept of device independence in very long-distance quantum communication experiments, which allows to reduce to the minimum of the trust assumptions on quantum devices in cryptographic scenarios, hence opening the way to global-scale secure communications. In the longer term, exploring the limits of QM would require going beyond the low-Earth orbit of ISS, and performing the gravitational decoherence and Bell tests on geostationary orbits. Significant developments for designing the necessary quantum payloads (including the entangled-photon source for the Bell test) would be necessary for such experiments.

The upcoming advent of nano spacecraft weighing a few grams and propelled via solar sails and vastly powerful ground-based lasers offers new opportunities to test fundamental quantum physics. Calculations show that such spacecraft can be accelerated to velocities >0.2 c with just a 10-minute burst of laser power from the earth or a lunar station. Creating the sails out of a nonlinear optical material or the payload consisting of a photonic chip would allow for the generation of entanglement and the whole host of fundamental tests that entanglement enables at these unprecedented velocities.

## Outlook and summary

In this paper, we present scientific and programmatic priorities to be considered by ESA, in some cases together with other space agencies, in view of an effective program towards the study of fundamental physics problems. The latter is related to tests of GR and their connection to QM as well as to the understanding of DM and DE. The proposed experiments make use of the space-based platforms currently available (ISS) or planned (Moon and Mars orbiters, landers, and rowers) in the Human and Robotic Exploration (HRE) program of ESA and microgravity platforms like sounding rockets, parabolic flights, and drop towers. The access to space provided by the HRE facilities allows to development of many interesting experiments. Following this review, a set of recommendations addressing the required technology development and setting priorities for a roadmap to space has been derived and presented in^183^. In a similar exercise, now limited to cold-atom sensors^184^, a technology development roadmap leading to space is also discussed.

Therefore, it is important that ESA starts a vigorous program on ultracold atoms in microgravity or in low-Earth orbit facilities. In the longer term, this will also allow to implementation of space-borne matter-wave interferometers, which can be used to test GR in the quantum regime and also to search for certain types of DM. Following the ACES mission, it is envisageable to develop optical atomic clocks for space missions, which would be important both for tests of GR and for relativistic geodesy. Improved lunar ranging experiments, using for instance optical links, would be highly effective to better probe some aspects of the EP as WEP and SEP. Radio and laser ranging experiments from Moon or Mars could put constraints on several alternative theories of gravity.

This domain of activities has the potential for many promising developments for progressing our knowledge in fundamental physics and fostering applications in several areas of research relevant to Earth science and exploration. Among them, we wish to recall applications in time & frequency metrology, universal time scales, reference systems, navigation, geodesy, and planet interior studies.

### Reporting summary

Further information on research design is available in the Nature Research Reporting Summary linked to this article.

## Supplementary information

Reporting Summary

## Data Availability

No data sets were generated or analyzed during the current study.

## References

[1] Capozziello, S. & Faraoni, V. *Beyond Einstein Gravity*, vol. 170. http://www.springerlink.com/content/hl1805/#section=801705&page=1 (Springer, Dordrecht, 2011).

[2] Misner, C. W., Thorne, K. S. & Wheeler, J. A. *Gravitation* (W. H. Freeman, San Francisco, 1973).

[3] Giulini, D. Equivalence principle, quantum mechanics, and atom-interferometric tests. https://arxiv.org/abs/1105.0749 (2012).

[4] Einstein, A. Grundgedanken und Methoden der Relativitätstheorie in ihrer Entwicklung dargestellt. In Janssen, M., Schulmann, R., Illy, J., Lehner, C. & Kormos Buchwald, D. (eds.) *The Collected Papers of Albert Einstein*, Vol. 7 (Princeton University Press, Princeton, 2002).

[5] Capozziello S, De Laurentis M (2011). Extended theories of gravity. Phys. Rep..

[6] Capozziello S, Francaviglia M (2008). Extended theories of gravity and their cosmological and astrophysical applications. Gen. Relativ. Gravit..

[7] Capozziello S, De Laurentis M (2012). The dark matter problem from f(R) gravity viewpoint. Annalen Phys..

[8] Blasone M, Capozziello S, Lambiase G, Petruzziello L (2019). Equivalence principle violation at finite temperature in scalar-tensor gravity. Eur. Phys. J. Plus.

[9] Bertone G, Hooper D, Silk J (2005). Particle dark matter: evidence, candidates and constraints. Phys. Rep..

[10] Strigari LE (2013). Galactic searches for dark matter. Phys. Rep..

[11] Magaña J, Matos T (2012). A brief review of the scalar field dark matter model. J. Phys. Conf. Ser..

[12] Graham PW, Kaplan DE, Mardon J, Rajendran S, Terrano WA (2016). Dark matter direct detection with accelerometers. Phys. Rev. D.

[13] Hees A, Guéna J, Abgrall M, Bize S, Wolf P (2016). Searching for an oscillating massive scalar field as a dark matter candidate using atomic hyperfine frequency comparisons. Phys. Rev. Lett..

[14] Arvanitaki A, Graham PW, Hogan JM, Rajendran S, Van Tilburg K (2018). Search for light scalar dark matter with atomic gravitational wave detectors. Phys. Rev. D.

[15] Elder B (2016). Chameleon dark enery and atom interferometry. Phys. Rev. D.

[16] Chiow S-w, Yu N (2020). Constraining symmetron dark energy using atom interferometry. Phys. Rev. D.

[17] Schrödinger E (1935). Die gegenwärtige situation in der quantenmechanik. Die Naturwissenschaften.

[18] Leggett AJ (2013). Macroscopic quantum systems and the quantum theory of measurement. Prog. Theor. Phys. Suppl..

[19] Ghirardi GC, Rimini A, Weber T (1986). Unified dynamics for microscopic and macroscopic systems. Phys. Rev. D.

[20] Weinberg S (1989). Precision tests of quantum mechanics. Phys. Rev. Lett..

[21] Penrose R (1996). On gravity’s role in quantum state reduction. Gen. Relativ. Gravit..

[22] Adler SL (2012). Quantum theory as an emergent phenomenon: foundations and phenomenology. J. Phys. Conf. Ser..

[23] Weinberg S (2012). Collapse of the state vector. Phys. Rev. A.

[24] Bassi A, Ghirardi G (2003). Dynamical reduction models. Phys. Rep..

[25] Bassi A, Lochan K, Satin S, Singh TP, Ulbricht H (2013). Models of wave-function collapse, underlying theories, and experimental tests. Rev. Mod. Phys..

[26] Bahrami M, Paternostro M, Bassi A, Ulbricht H (2014). Proposal for a noninterferometric test of collapse models in optomechanical systems. Phys. Rev. Lett..

[27] Carlesso M, Bassi A, Falferi P, Vinante A (2016). Experimental bounds on collapse models from gravitational wave detectors. Phys. Rev. D.

[28] Vinante A, Mezzena R, Falferi P, Carlesso M, Bassi A (2017). Improved noninterferometric test of collapse models using ultracold cantilevers. Phys. Rev. Lett..

[29] Piscicchia K (2017). CSL collapse model mapped with the spontaneous radiation. Entropy.

[30] Vinante A (2020). Narrowing the parameter space of collapse models with ultracold layered force sensors. Phys. Rev. Lett..

[31] Donadi S (2020). Underground test of gravity-related wave function collapse. Nat. Phys.

[32] Touboul P (2017). Microscope mission: first results of a space test of the equivalence principle. Phys. Rev. Lett..

[33] Cacciapuoti L (2020). Testing gravity with cold-atom clocks in space. Eur. Phys. J. D.

[34] Aguilera DN (2014). STE-QUEST—test of the universality of free fall using cold atom interferometry. Class. Quantum Gravity.

[35] Tino GM (2019). SAGE: a proposal for a space atomic gravity explorer. Eur. Phys. J. D.

[36] Becker D (2018). Space-borne Bose–Einstein condensation for precision interferometry. Nature.

[37] Altschul B (2015). Quantum tests of the Einstein equivalence principle with the STE-QUEST space mission. Adv. Space Res..

[38] Delva P (2018). Gravitational redshift test using eccentric Galileo satellites. Phys. Rev. Lett..

[39] Herrmann S (2018). Test of the gravitational redshift with Galileo satellites in an eccentric orbit. Phys. Rev. Lett..

[40] Kostelecký VA, Tasson JD (2011). Matter-gravity couplings and Lorentz violation. Phys. Rev. D.

[41] Merkowitz SM (2010). Tests of gravity using lunar laser ranging. Living Rev. Relativ..

[42] Bertotti B, Iess L, Tortora P (2003). A test of general relativity using radio links with the Cassini spacecraft. Nature.

[43] Imperi L, Iess L, Mariani MJ (2018). An analysis of the geodesy and relativity experiments of BepiColombo. Icarus.

[44] Xu P (2019). Satellite testing of a gravitationally induced quantum decoherence model. Science.

[45] Wolf P, Alonso R, Blas D (2019). Scattering of light dark matter in atomic clocks. Phys. Rev. D.

[46] Leanhardt AE (2003). Cooling Bose-Einstein condensates below 500 picokelvin. Science.

[47] Aveline DC (2020). Observation of Bose–Einstein condensates in an Earth-orbiting research lab. Nature.

[48] Carollo RA (2022). Observation of ultracold atomic bubbles in orbital microgravity. Nature.

[49] Frye K (2021). The Bose-Einstein condensate and cold atom laboratory. EPJ Quantum Technol..

[50] Lipa JA, Nissen JA, Stricker DA, Swanson DR, Chui TCP (2003). Specific heat of liquid helium in zero gravity very near the lambda point. Phys. Rev. B.

[51] Bilardello M, Donadi S, Vinante A, Bassi A (2016). Bounds on collapse models from cold-atom experiments. Phys. A: Stat. Mech. Appl..

[52] Ghirardi GC, Pearle P, Rimini A (1990). Markov processes in Hilbert space and continuous spontaneous localization of systems of identical particles. Phys. Rev. A.

[53] Bilardello M, Trombettoni A, Bassi A (2017). Collapse in ultracold Bose Josephson junctions. Phys. Rev. A.

[54] Kovachy T (2015). Matter wave lensing to picokelvin temperatures. Phys. Rev. Lett..

[55] McGrew WF (2018). Atomic clock performance enabling geodesy below the centimetre level. Nature.

[56] Petit G (2015). 1E-16 frequency transfer by GPS PPP with integer ambiguity resolution. Metrologia.

[57] Bodine MI (2020). Optical atomic clock comparison through turbulent air. Phys. Rev. Research.

[58] Lisdat C (2016). A clock network for geodesy and fundamental science. Nat. Commun..

[59] Sanner C (2019). Optical clock comparison for Lorentz symmetry testing. Nature.

[60] Smorra C (2017). A parts-per-billion measurement of the antiproton magnetic moment. Nature.

[61] Smorra C (2019). Direct limits on the interaction of antiprotons with axion-like dark matter. Nature.

[62] Cronin AD, Schmiedmayer J, Pritchard DE (2009). Optics and interferometry with atoms and molecules. Rev. Mod. Phys..

[63] Tino, G. M. & Kasevich, M. A. (eds.). *Atom Interferometry*, vol. 188 of *Proceedings of the International School of Physics "Enrico Fermi”* (IOS Press, Amsterdam, 2014).

[64] Kleinert S, Kajari E, Roura A, Schleich WP (2015). Representation-free description of light-pulse atom interferometry including noninertial effects. Phys. Rep..

[65] Bongs K (2019). Taking atom interferometric quantum sensors from the laboratory to real-world applications. Nat. Rev. Phys..

[66] Asenbaum P, Overstreet C, Kim M, Curti J, Kasevich MA (2020). Atom-interferometric test of the equivalence principle at the 10^−12^ level. Phys. Rev. Lett..

[67] Schlippert D (2014). Quantum test of the universality of free fall. Phys. Rev. Lett..

[68] Barrett B (2016). Dual matter-wave inertial sensors in weightlessness. Nat. Commun..

[69] Battelier B (2021). Exploring the foundations of the universe with space tests of the equivalence principle. Exp. Astron..

[70] Williams J, Chiow S-w, Yu N, Müller H (2016). Quantum test of the equivalence principle and space-time aboard the international space station. New J. Phys..

[71] Roura A (2017). Circumventing Heisenberg’s uncertainty principle in atom interferometry tests of the equivalence principle. Phys. Rev. Lett..

[72] D’Amico G (2017). Canceling the gravity gradient phase shift in atom interferometry. Phys. Rev. Lett..

[73] Overstreet C (2018). Effective inertial frame in an atom interferometric test of the equivalence principle. Phys. Rev. Lett..

[74] Loriani, S. et al. Resolution of the co-location problem in satellite quantum tests of the universality of free fall. https://arxiv.org/abs/2006.08729. (2020).

[75] Tino G (2013). Precision gravity tests with atom interferometry in space. Nucl. Phys. B Proc. Suppl..

[76] Hamilton P (2015). Atom-interferometry constraints on dark energy. Science.

[77] Jaffe M (2017). Testing sub-gravitational forces on atoms from a miniature in-vacuum source mass. Nat. Phys..

[78] Sabulsky DO (2019). Experiment to detect dark energy forces using atom interferometry. Phys. Rev. Lett..

[79] Chiow S-w, Yu N (2018). Multiloop atom interferometer measurements of chameleon dark energy in microgravity. Phys. Rev. D.

[80] Hogan JM, Kasevich MA (2016). Atom-interferometric gravitational-wave detection using heterodyne laser links. Phys. Rev. A.

[81] Graham PW, Hogan JM, Kasevich MA, Rajendran S (2013). New method for gravitational wave detection with atomic sensors. Phys. Rev. Lett..

[82] Graham, P. W., Hogan, J. M., Kasevich, M. A., Rajendran, S. & Romani, R. W. Mid-band gravitational wave detection with precision atomic sensors. https://arxiv.org/abs/1711.02225. (2017). 10.1103/PhysRevLett.110.171102 23679702

[83] El-Neaj YA (2020). AEDGE: atomic experiment for dark matter and gravity exploration in space. EPJ Quantum Technol..

[84] Origlia S (2018). Towards an optical clock for space: compact, high-performance optical lattice clock based on bosonic atoms. Phys. Rev. A.

[85] Roura A (2020). Gravitational redshift in quantum-clock interferometry. Phys. Rev. X.

[86] Rosi G (2017). Quantum test of the equivalence principle for atoms in coherent superposition of internal energy states. Nat. Commun..

[87] Aspelmeyer M, Kippenberg TJ, Marquardt F (2014). Cavity optomechanics. Rev. Mod. Phys..

[88] Fein YY (2019). Quantum superposition of molecules beyond 25 kda. Nat. Phys..

[89] Arvanitaki A, Geraci AA (2013). Detecting high-frequency gravitational waves with optically levitated sensors. Phys. Rev. Lett..

[90] Pontin A, Mourounas LS, Geraci AA, Barker PF (2018). Levitated optomechanics with a fiber fabry–perot interferometer. New J. Phys..

[91] Qvarfort S, Serafini A, Barker PF, Bose S (2018). Gravimetry through non-linear optomechanics. Nat. Commun..

[92] Hebestreit E, Frimmer M, Reimann R, Novotny L (2018). Sensing static forces with free-falling nanoparticles. Phys. Rev. Lett..

[93] Carney D (2020). Mechanical quantum sensing in the search for dark matter. Quantum Sci. Technol.

[94] Carney, D., Hook, A., Liu, Z., Taylor, J. M. & Zhao, Y. Ultralight dark matter detection with mechanical quantum sensors. https://arxiv.org/abs/1908.04797 (2019).

[95] Rider AD (2016). Search for screened interactions associated with dark energy below the 100 μm length scale. Phys. Rev. Lett.

[96] Carlesso M, Bassi A, Paternostro M, Ulbricht H (2019). Testing the gravitational field generated by a quantum superposition. New J. Phys..

[97] Carlesso, M., Paternostro, M., Ulbricht, H. & Bassi, A. When Cavendish meets Feynman: a quantum torsion balance for testing the quantumness of gravity. https://arxiv.org/abs/1710.08695 (2017).

[98] Fadeev, P. et al. Gravity probe spin: prospects for measuring general-relativistic precession of intrinsic spin using a ferromagnetic gyroscope. https://arxiv.org/abs/2006.09334 (2020).

[99] Riedel CJ (2013). Direct detection of classically undetectable dark matter through quantum decoherence. Phys. Rev. D.

[100] Bateman J, McHardy I, Merle A, Morris TR, Ulbricht H (2015). On the existence of low-mass dark matter and its direct detection. Sci. Rep..

[101] Riedel CJ, Yavin I (2017). Decoherence as a way to measure extremely soft collisions with dark matter. Phys. Rev. D.

[102] Kaltenbaek R (2016). Macroscopic quantum resonators (maqro): 2015 update. EPJ Quantum Technol..

[103] Kaltenbaek, R. Tests in space. In *Do Wave Functions Jump?* 401–411 (Springer).

[104] Bateman J, Nimmrichter S, Hornberger K, Ulbricht H (2014). Near-field interferometry of a free-falling nanoparticle from a point-like source. Nat. Commun..

[105] Belenchia A, Gasbarri G, Kaltenbaek R, Ulbricht H, Paternostro M (2019). Talbot-Lau effect beyond the point-particle approximation. Phys. Rev. A.

[106] Wan C (2016). Free nano-object Ramsey interferometry for large quantum superpositions. Phys. Rev. Lett..

[107] Stickler BA (2018). Probing macroscopic quantum superpositions with nanorotors. New J. Phys..

[108] Carlesso M, Paternostro M, Ulbricht H, Vinante A, Bassi A (2018). Non-interferometric test of the continuous spontaneous localization model based on rotational optomechanics. New J. Phys..

[109] Millen, J. & Stickler, B. A. Quantum experiments with microscale particles. https://arxiv.org/abs/2010.07641 (2020).

[110] Grossardt A, Bateman J, Ulbricht H, Bassi A (2016). Optomechanical test of the Schrödinger-Newton equation. Phys. Rev. D.

[111] Bassi A, Grossardt A, Ulbricht H (2017). Gravitational decoherence. Class. Quantum Grav..

[112] Bahrami M, Smirne A, Bassi A (2014). Role of gravity in the collapse of a wave function: a probe into the diósi-penrose model. Phys. Rev. A.

[113] Penrose R (2014). On the gravitization of quantum mechanics 2: conformal cyclic cosmology. Found. Phys.

[114] Diósi L (1989). Models for universal reduction of macroscopic quantum fluctuations. Phys. Rev. A.

[115] Hu BL, Verdaguer E (2008). Stochastic gravity: theory and applications. Living Rev. Relativ..

[116] Hu BL, Roura A, Verdaguer E (2004). Induced quantum metric fluctuations and the validity of semiclassical gravity. Phys. Rev. D.

[117] Roura A, Verdaguer E (2008). Cosmological perturbations from stochastic gravity. Phys. Rev. D.

[118] Fröb MB, Roura A, Verdaguer E (2012). One-loop gravitational wave spectrum in dark energy Sitter spacetime. J. Cosmol. Astropart. Phys..

[119] Bose S (2017). Spin entanglement witness for quantum gravity. Phys. Rev. Lett..

[120] Belenchia A (2018). Quantum superposition of massive objects and the quantization of gravity. Phys. Rev. D.

[121] Belenchia A (2016). Testing quantum gravity induced nonlocality via optomechanical quantum oscillators. Phys. Rev. Lett..

[122] Belenchia A (2019). Tests of quantum gravity-induced non-locality: Hamiltonian formulation of a non-local harmonic oscillator. Class. Quantum Grav..

[123] Pikovski I, Zych M, Costa F, Brukner Č (2015). Universal decoherence due to gravitational time dilation. Nat. Phys..

[124] Toroš, M., Grossardt, A. & Bassi, A. Quantum mechanics for non-inertial observers. https://arxiv.org/abs/1701.04298 (2017).

[125] Giulini, D., Großardt, A. & Schwartz, P. K. Coupling Quantum Matter and Gravity. *General Relativity and Quantum Cosmology*. 10.48550/arXiv.2207.05029 (2022).

[126] Fink M (2017). Experimental test of photonic entanglement in accelerated reference frames. Nat. Commun..

[127] Restuccia S (2019). Photon bunching in a rotating reference frame. Phys. Rev. Lett..

[128] Toroš M (2020). Revealing and concealing entanglement with noninertial motion. Phys. Rev. A.

[129] Bose S (2017). Spin entanglement witness for quantum gravity. Phys. Rev. Lett..

[130] Marletto C, Vedral V (2017). Gravitationally induced entanglement between two massive particles is sufficient evidence of quantum effects in gravity. Phys. Rev. Lett..

[131] Bender P (1973). The lunar laser ranging experiment. Science.

[132] Dell’Agnello, S. et al. LaRRI: laser retro-reflector for InSight Mars lander. *Space Res. Today* **200**, 25–32 (2017).

[133] Porcelli L (2019). Optical-performance testing of the laser retroreflector for InSight. Space Sci. Rev..

[134] Banerdt W, Russell C (2017). Topical collection on InSight mission to Mars. Space Sci. Rev..

[135] Degnan, J. J. Asynchronous laser transponders: anew tool for improved fundamental physics experiments. In: *From Quantum to Cosmos*, 265–278. 10.1142/9789814261210_0021 (World Scientific, 2009).

[136] Mao D (2016). The laser ranging experiment of the lunar reconnaissance orbiter: five years of operations and data analysis. Icarus.

[137] Robinson, B. et al. The NASA Lunar Laser Communication Demonstration - successful high-rate laser communications to and from the Moon. In: *SpaceOps Conference* (American Institute of Aeronautics and Astronautics (USA), 2014).

[138] Mazarico E (2020). First two-way laser ranging to a lunar orbiter: infrared observations from the Grasse station to LRO’s retro-reflector array. Earth Planets Space.

[139] Fienga A, Laskar J, Exertier P, Manche H, Gastineau M (2015). Numerical estimation of the sensitivity of inpop planetary ephemerides to general relativity parameters. Celest. Mech. Dyn. Astron..

[140] Smith DE (2018). Trilogy, a planetary geodesy mission concept for measuring the expansion of the solar system. Planet. Space Sci..

[141] Williams J (2004). Progress in lunar laser ranging tests of relativistic gravity. Phys. Rev. Lett..

[142] Martini, M. & Dell’Agnello, S. Probing gravity with next generation lunar laser ranging. In *Gravity: Where Do We Stand?* 195–210 (Springer International Publishing (Switzerland), 2016).

[143] Currie, D. et al. A Lunar Laser Ranging Retroreflector Array for the 21st Century. In: *IV International Conference on Particle and Fundamental Physics in Space*, vol. 243–244, 218–228 (Elsevier B.V., 2013).

[144] March R (2011). Constraining spacetime torsion with the Moon and Mercury. Phys. Rev. D.

[145] March R (2011). Constraining spacetime torsion with LAGEOS. Gen. Relativ. Gravit..

[146] Bertolami O (2013). Solar system constraints to nonminimally coupled gravity. Phys. Rev. D.

[147] March R (2013). 1/c expansion of nonminimally coupled curvature-matter gravity models and constraints from planetary precession. Phys. Rev. D.

[148] Williams J (2006). Lunar laser ranging science: Gravitational physics and lunar interior and geodesy. Adv. Space Res..

[149] Weber R (2011). Seismic detection of the lunar core. Science.

[150] Neal, C. et al. The Lunar Geophysical Network mission. In *50th Lunar and Planetary Science Conference*, 2132 (Lunar and Planetary Institute (USA), 2019).

[151] Ciocci E (2017). Performance analysis of next-generation lunar laser retroreflectors. Adv. Space Res..

[152] Shapiro I (1988). Measurement of the de Sitter precession of the Moon: a relativistic three-body effect. Phys. Rev. Lett..

[153] Dell’Agnello S (2017). INRRI-EDM/2016: the first laser retroreflector on the surface of Mars. Adv. Space Research.

[154] Dell’Agnello, S. et al. Laser retroreflectors for InSight and an international Mars Geophysical Network (MGN). In *50th Lunar and Planetary Science Conference*, 1492 (Lunar and Planetary Institute (USA), 2019).

[155] NASA-ESA Mars Sample Return program: missions and spacecrafts description (2019). www.esa.int/Science_Exploration/Human_and_Robotic_Exploration/Exploration/Mars_sample_return (2019).

[156] NASA-ESA Mars Sample Return: Independent Review Board recommendations (2020). www.nasa.gov/sites/default/files/atoms/files/nasa_esa_mars_sample_return_irb_report.pdf (2020).

[157] Dell’Agnello, S. et al. Testing gravity with the Moon and Mars. In: *Vulcano Workshop on Frontier Objects in Astrophysics and Particle Physics*, vol. 66, 294–301. http://www.lnf.infn.it/sis/frascatiseries/Volume66/Volume66.pdf (Frascati Physics Series (Italy), 2018).

[158] Crosta, M. Testing gravity with GAIA. In: *Vulcano Workshop on Frontier Objects in Astrophysics and Particle Physics*, vol. 66, 302–311. http://www.lnf.infn.it/sis/frascatiseries/Volume66/Volume66.pdf (Frascati Physics Series (Italy), 2018).

[159] Joshi SK (2018). Space Quest mission proposal: experimentally testing decoherence due to gravity. New J. Phys..

[160] Mohageg, M. et al. The Deep Space Quantum Link: Prospective Fundamental Physics Experiments using Long-Baseline *Quantum Opt.* https://arxiv.org/abs/2111.15591 (2021). 10.1140/epjqt/s40507-022-00143-0 PMC9547810 36227029

[161] Ralph TC, Milburn GJ, Downes T (2009). Quantum connectivity of space-time and gravitationally induced decorrelation of entanglement. Phys. Rev. A.

[162] Ralph TC, Pienaar J (2014). Entanglement decoherence in a gravitational well according to the event formalism. New J. Phys..

[163] Zych M, Costa F, Pikovski I, Ralph TC, Brukner Č (2012). General relativistic effects in quantum interference of photons. Class. Quantum Gravit.

[164] Colella R, Overhauser AW, Werner SA (1975). Observation of gravitationally induced quantum interference. Phys. Rev. Lett..

[165] Brodutch A, Gilchrist A, Guff T, Smith ARH, Terno DR (2015). Post-Newtonian gravitational effects in optical interferometry. Phys. Rev. D.

[166] Pallister S (2017). A blueprint for a simultaneous test of quantum mechanics and general relativity in a space-based quantum optics experiment. EPJ Quantum Technol..

[167] Roberts BM, Blewitt G, Dailey C, Derevianko A (2018). Search for transient ultralight dark matter signatures with networks of precision measurement devices using a Bayesian statistics method. Phys. Rev. D.

[168] Khoury J, Weltman A (2004). Chameleon fields: awaiting surprises for tests of gravity in space. Phys. Rev. Lett..

[169] Buoninfante L, Lambiase G, Stabile A (2020). Testing fundamental physics with photon frequency shift. Eur. Phys. J. C.

[170] Rideout D (2012). Fundamental quantum optics experiments conceivable with satellites-reaching relativistic distances and velocities. Class. Quantum Gravit..

[171] Vallone G (2016). Interference at the single photon level along satellite-ground channels. Phys. Rev. Lett..

[172] Bruschi DE, Ralph TC, Fuentes I, Jennewein T, Razavi M (2014). Spacetime effects on satellite-based quantum communications. Phys. Rev. D.

[173] Kohlrus J, Bruschi DE, Louko J, Fuentes I (2017). Quantum communications and quantum metrology in the spacetime of a rotating planet. EPJ Quantum Technol..

[174] Liu T, Cao S, Wu S, Zeng H (2019). The influence of the earth’s curved spacetime on gaussian quantum coherence. Laser Phys. Lett..

[175] Wu S-M, Li Z-C, Zeng H-S (2020). Quantum coherence of multipartite w-state in a schwarzschild spacetime. Europhys. Lett..

[176] Dailey, C. et al. Quantum sensor networks as exotic field telescopes for multi-messenger astronomy. https://www.nature.com/articles/s41550-020-01242-7 (2020).

[177] Curtin D, Setford J (2020). How to discover mirror stars. Phys. Lett. B.

[178] Penrose R (1996). On gravity’s role in quantum state reduction. Gen. Relativ. Gravit..

[179] Kafri D, Taylor JM, Milburn GJ (2014). A classical channel model for gravitational decoherence. New J. Phys..

[180] Deutsch D (1991). Quantum mechanics near closed timelike lines. Phys. Rev. D.

[181] Pienaar JL, Ralph TC, Myers CR (2013). Open timelike curves violate Heisenberg’s uncertainty principle. Phys. Rev. Lett..

[182] Scheidl T, Wille E, Ursin R (2013). Quantum optics experiments using the international space station: a proposal. New J. Phys..

[183] Bassi, A. et al. White Paper 01: Fundamental Physics. https://esamultimedia.esa.int/docs/HRE/01_PhysicalSciences_Fundamental_Physics.pdf (2021).

[184] Alonso, I. et al. Cold Atoms in Space: Community Workshop Summary and Proposed Road-Map. 10.48550/arXiv.2201.07789 (2022).

[185] De Marchi, F. & Cascioli, G. Testing general relativity in the solar system: present and future perspectives. *Class. Quantum Gravity* https://arxiv.org/abs/1911.05561 (2020).

[186] Williams, J. et al. Lunar Laser Ranging on Artemis III: Operation and Science Goals. *White Paper submitted to the Science Definition Team of the Artemis III mission of NASA*. https://www.lpi.usra.edu/announcements/artemis/whitepapers/2073.pdf (2020).

[187] ESA Strategy for Science at the Moon. https://exploration.esa.int/web/moon/ (2019).

[188] ESA European Large Logistics Lander for the Moon. https://ideas.esa.int/servlet/hype/IMT?documentTableId=45087607022749715&userAction=Browse&templateName=&documentId=7257e8d165a32b877027ebfc5e67d8d9 (2020).

